# The Dynamic Endothelial Activation and Stress Index (EASIX) as a Predictor of Early Death and Long-Term Survival in Acute Promyelocytic Leukemia (APL): A Multicenter Study

**DOI:** 10.3390/cancers18050843

**Published:** 2026-03-05

**Authors:** Fazıl Çağrı Hunutlu, Vildan Özkocaman, Mehmet Baysal, Hikmet Öztop, Saide Elif Güllülü Boz, Nevriye Gül Ada Tak, Oğuzhan Sertkaya, İlknur Kara, Emre Akar, Şüheda Çakmak, Ahmet Mert Yanık, Tuba Güllü Koca, İbrahim Ethem Pınar, Vildan Gürsoy, Tuba Ersal, Seval Akpınar, Yusuf Bilen, Fahir Özkalemkaş

**Affiliations:** 1 Division of Hematology, Department of Internal Medicine, University of Health Sciences, Bursa Sehir Training and Research Hospital, 16110 Bursa, Turkey; ahmet.mert@marmara.edu.tr (A.M.Y.); tubagullukoca@uludag.edu.tr (T.G.K.); yusuf.bilen@saglik.gov.tr (Y.B.); 2 Division of Hematology, Department of Internal Medicine, Faculty of Medicine, Bursa Uludag University, 16059 Bursa, Turkey; vildanoz@uludag.edu.tr (V.Ö.); iethempinar@uludag.edu.tr (İ.E.P.); vildangursoy@uludag.edu.tr (V.G.); tubaersal@uludag.edu.tr (T.E.); fahir@uludag.edu.tr (F.Ö.); 3 Division of Hematology, Department of Internal Medicine, Faculty of Medicine, Tekirdag Namik Kemal University, 59030 Tekirdag, Turkey; mbaysal@nku.edu.tr (M.B.); eakar@nku.edu.tr (E.A.); scakmak@nku.edu.tr (Ş.Ç.); seakpinar@nku.edu.tr (S.A.); 4 Department of Internal Medicine, Faculty of Medicine, Bursa Uludag University, 16059 Bursa, Turkey; hikmetoztop@uludag.edu.tr (H.Ö.); ngada@uludag.edu.tr (N.G.A.T.); oguzhansertkaya@uludag.edu.tr (O.S.); 5 Division of Nephrology, Department of Internal Medicine, University of Health Sciences, Bursa Yüksek Ihtisas Training and Research Hospital, 16310 Bursa, Turkey; elifgullulu@uludag.edu.tr; 6 Department of Internal Medicine, Faculty of Medicine, Tekirdag Namik Kemal University, 59030 Tekirdag, Turkey; ikara@nku.edu.tr

**Keywords:** acute promyelocytic leukemia (APL), endothelial activation and stress index (EASIX), early death (ED), dynamic risk stratification, AIDA protocol

## Abstract

**Background/Objectives:** Early death (ED) remains the primary barrier to long-term survival in acute promyelocytic leukemia (APL). Since current risk stratification models rely solely on static baseline parameters, they fail to capture the high biological volatility during the induction phase. We aimed to evaluate the prognostic value of the dynamic evolution of the endothelial activation and stress index (EASIX). **Materials and Methods:** This multicenter, retrospective study analyzed 131 newly diagnosed adult APL patients treated with the AIDA protocol. EASIX scores were calculated at admission (D0) and day 7 (D7). ROC, landmark, multivariable logistic and Cox regression analyses were performed to assess the impact of dynamic endothelial changes (ΔEASIX) on mortality and survival. **Results:** The ED rate was 25.2%. While baseline EASIX successfully predicted very early death (<7 days), dynamic assessment provided superior risk stratification. Worsening endothelial status (ΔEASIX > 0.35) was an independent predictor of early mortality (OR: 12.41, *p* = 0.007), inferior EFS (HR: 5.70, *p* = 0.004), and poor OS (HR: 3.69, *p* = 0.023). Landmark analysis stratified by the kinetic trajectory of ∆EASIX confirmed that patients above the optimal cut-off had significantly inferior 3-year EFS (56.3% vs. 77.8%, *p* = 0.041) and OS (59.5% vs. 78.0%, *p* = 0.026). **Conclusions:** To our knowledge, this is the first study to establish EASIX as a dynamic prognostic marker in APL. Our findings indicate that the “kinetic trajectory” of endothelial stress is a more accurate predictor of survival than static baseline assessment alone. While dynamic EASIX monitoring offers a valuable tool for real-time risk reclassification, these results require validation through prospective studies.

## Simple Summary

Acute promyelocytic leukemia is a highly curable leukemia subtype; however, early mortality driven by severe coagulopathy and vascular injury remains a significant clinical challenge. Traditional risk models rely solely on static measurements taken at diagnosis, failing to capture the rapid physiological changes that occur during the initial phase of treatment. This study addressed this gap by evaluating the dynamic evolution of the endothelial activation and stress index, a composite marker of vascular stress, over the first week of therapy. We demonstrated that patients experiencing a deterioration in endothelial function faced a drastically increased risk of early death, whereas those who achieved stabilization exhibited excellent survival outcomes. These findings offer clinicians a practical, real-time tool for identifying high-risk patients who require intensified supportive care, thereby providing a new strategy to reduce early mortality and improve long-term prognosis.

## 1. Introduction

Acute promyelocytic leukemia (APL) is a distinct subtype of acute myeloid leukemia (AML) characterized by the *PML::RARA* fusion gene resulting from the t(15;17)(q22;q21) translocation, which induces maturation arrest at the promyelocytic stage [1]. Historically considered the most fatal AML subtype due to severe coagulopathy, the introduction of all-trans retinoic acid (ATRA) and arsenic trioxide (ATO) has transformed APL into a highly curable disease with long-term cure rates exceeding 90% in the current era [2,3]. Despite these remarkable therapeutic advances, early death (ED) remains the primary barrier to long-term survival persisting as the major cause of treatment failure [4].

ED is generally defined as death from any cause within the first 30 days of diagnosis. While clinical trials report ED rates of 2–6%, real-world data reveal significantly higher rates reaching up to 37% [5,6,7,8]. A substantial proportion of these fatalities occur within the first week of diagnosis, termed “very early death” (VED); however, data regarding the incidence and specific risk factors for VED remain limited [9,10,11]. While intracranial hemorrhage, disseminated intravascular coagulation (DIC), infections and differentiation syndrome (DS) are identified as the leading causes of ED, standard risk stratification models are primarily designed to predict relapse rather than early mortality [6,12,13].

Recently, novel risk scores developed by Österroos et al. and Cai et al. have demonstrated superior performance over the Sanz score in predicting ED and have been validated in independent cohorts [6,14]. While these models incorporate specific baseline variables, numerous studies have broadly identified advanced age, high white blood cell (WBC) count, thrombocytopenia, poor Eastern Cooperative Oncology Group (ECOG) performance status, and elevated lactate dehydrogenase (LDH) and creatinine levels as key predictors of early mortality [1,9,15]. However, these risk stratification systems rely exclusively on static baseline parameters obtained at admission. Given the rapid physiological changes induced by induction therapy, static markers may fail to capture the dynamic evolution of endothelial injury, coagulopathy, DS and infectious complications during the critical first month of treatment.

The endothelial activation and stress index (EASIX), calculated using creatinine, LDH and platelet counts, serves as a validated surrogate marker for endothelial activation and stress in both benign and malignant conditions. High baseline EASIX scores have been correlated with poor survival in multiple myeloma and Hodgkin and non-Hodgkin lymphoma [16,17,18,19,20,21,22]. Particularly in allogeneic hematopoietic stem cell transplantation (allo-HSCT), where endothelial damage is prominent, the dynamic monitoring of EASIX scores has been associated with inferior survival and increased microvascular complications [23]. This prognostic utility also extends to immune-mediated thrombotic thrombocytopenic purpura (iTTP), a disorder characterized by intravascular microthrombosis. In these patients, elevated EASIX scores at diagnosis and during follow-up are strongly linked to poorer treatment responses and increased relapse rates [24].

Despite the evolution of treatment modalities in APL, ED remains a critical clinical challenge. While scoring systems exist to determine ED risk at diagnosis, there is currently no dynamic scoring system capable of identifying which patients experience increased mortality risk and which patients approach a safer range during treatment. In this study, recognizing endothelial dysfunction as a key driver of APL-associated mortality, we aimed to assess the prognostic value of baseline EASIX scores and their dynamic changes during early induction for ED and long-term survival.

## 2. Materials and Methods

### 2.1. Study Design and Patients

**Figure 1:**
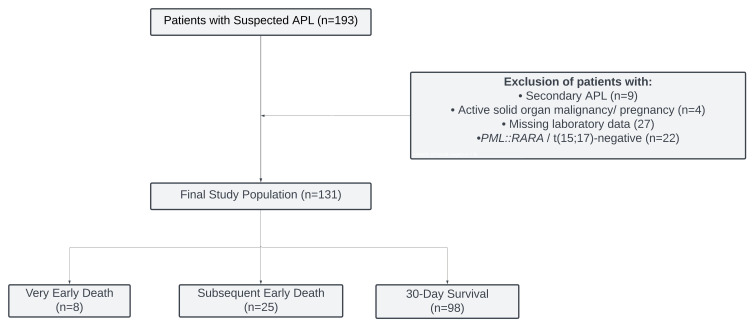
Flowchart of this study.

This multicenter, retrospective cohort study analyzed data from adult patients consecutively diagnosed with APL at two tertiary referral centers in Turkey (Bursa Uludag University and Tekirdag Namik Kemal University) between January 2008 and May 2025. The study population comprised adult patients (aged ≥ 18 years) with de novo APL, diagnosed according to the World Health Organization (WHO) classification criteria [25]. The diagnosis was genetically verified by detecting the *PML::RARA* fusion transcript via a reverse transcriptase polymerase chain reaction (RT-PCR) or by the t(15;17) translocation using fluorescence in situ hybridization (FISH). Exclusion criteria encompassed cases with unconfirmed *PML::RARA* or t(15;17) status, secondary APL, concomitant hematological disorders, other active malignancies necessitating treatment and pregnancy. Figure 1 illustrates the patient selection flowchart. For the final cohort of 131 patients, comprehensive data regarding demographic information, clinicopathological characteristics, laboratory parameters, molecular profiles, risk stratification scores, therapeutic responses and survival outcomes were retrospectively extracted from medical records.

### 2.2. Treatment Regimens and Supportive Care

Due to national reimbursement policies restricting the use of ATO in the frontline setting, all patients received the standard AIDA protocol. Oral ATRA, 45 mg/m^2^/day, was initiated immediately upon clinical suspicion and continued until complete remission. Concurrent chemotherapy consisted of idarubicin (12 mg/m^2^) administered intravenously on days 2, 4, 6 and 8. Aggressive supportive care was instituted to manage coagulopathy. Transfusion thresholds were set to maintain platelet counts ≥ 30 × 10^9^/L, fibrinogen levels ≥ 150 mg/dL (via fresh frozen plasma or fibrinogen concentrate), and hemoglobin levels ≥ 8 g/dL. For prophylaxis against DS, intravenous dexamethasone (10 mg every 12 h) was administered for the first 5–10 days in high-risk patients (WBC ≥ 10 × 10^9^/L) lacking active infection. Upon the development of DS signs or symptoms, ATRA was temporarily withheld, and high-dose dexamethasone was continued until symptom resolution. Patients achieving complete remission proceeded to three cycles of anthracycline–ATRA-based consolidation. Maintenance therapy was continued for two years, comprising intermittent ATRA (45 mg/m^2^/day for 15 days every 3 months), daily 6-mercaptopurine (50 mg/m^2^) and weekly methotrexate (15 mg/m^2^) [26,27,28].

### 2.3. Definitions and Risk Stratification

Baseline risk stratification utilized the Sanz [29] and International Society of Thrombosis and Haemostasis (ISTH) scores [30], while the Österroos (incorporating age, WBC, platelets) [6] and Cai models (integrating age, WBC, platelets, LDH) [14] were applied specifically for ED risk prediction. ED was defined as mortality from any cause within the first 30 days of diagnosis, whereas VED referred to mortality occurring in less than 7 days (<7 days) of diagnosis. DS was diagnosed according to the criteria established by Frankel et al. [31], based on the presence of characteristic signs, including unexplained fever, weight gain (>5 kg), respiratory distress, pulmonary infiltrates, pleural/pericardial effusion, hypotension, and acute renal failure.

The EASIX score was computed using the standard formula: [LDH (U/L) × Creatinine (mg/dL)]/Platelet Count (10^9^/L) [32]. To address the skewed distribution of the raw EASIX score, a binary logarithm (Log_2_) transformation was applied [33]. We evaluated the baseline score (EASIX D0), the score on day 7 (EASIX D7) and the dynamic change (ΔEASIX), calculated as the day 7 score minus the baseline (D0) score. Day 7 was specifically selected as the temporal landmark for dynamic assessment for two primary reasons. Clinically, VED in APL is standardly defined as mortality within the first 7 days; utilizing day 7 allows for a robust landmark analysis to evaluate subsequent early mortality. Therapeutically, because the AIDA protocol involves anthracycline administration on days 2, 4, 6 and 8, day 7 effectively captures the peak cumulative endothelial damage near the completion of the active cytotoxic phase.

Overall survival (OS) was measured from diagnosis to death from any cause or last follow-up. Event-free survival (EFS) was calculated from diagnosis to the first event (relapse, death or last follow-up).

### 2.4. Statistical Analysis

Statistical analyses were performed using IBM SPSS Statistics version 29.0 (IBM Corp., Armonk, NY, USA) and R software (version 4.5.2; R Foundation for Statistical Computing, Vienna, Austria) within the RStudio (version 2026.01.0+392) environment. Data normality was assessed via the Shapiro–Wilk test. Continuous variables were presented as the mean ± standard deviation or median (min-max), while categorical variables were summarized as frequencies and percentages. Group comparisons utilized the Mann–Whitney U, one-way ANOVA or Kruskal–Wallis tests for continuous variables and the Chi-square or Fisher–Freeman–Halton tests for categorical data, depending on distribution and cell counts.

Prognostic cut-offs for EASIX scores (D0 for VED; D7 and ΔEASIX for mortality between days 7 and 30) were determined using receiver operating characteristic (ROC) curve analysis and the Youden index. To mitigate immortal time bias, a 7-day landmark analysis was applied for dynamic markers (D7/ΔEASIX), excluding VED (<7 days).

In this landmark cohort, survival probabilities (OS/EFS) were estimated via the landmark Kaplan–Meier method and compared using the Log-rank test. Variables yielding a *p*-value < 0.20 in univariate analysis were entered into the multivariate binary logistic regression (for 7–30 day mortality) and Cox proportional hazards regression (for OS/EFS) models. These multivariate models were constructed using the Backward stepwise (Likelihood Ratio) method to identify independent prognostic factors. Advanced visualizations (Sankey diagrams, violin plots) and model performance metrics (Net Reclassification Improvement [NRI]) were generated using specific R (version 4.5.2) packages (survival, survminer, ggalluvial, ggplot2, nricens). All tests were two-sided with a significance level of *p* < 0.05.

## 3. Results

### 3.1. Baseline Characteristics and Patient Profile

The baseline demographic and clinicopathological characteristics of the study cohort are summarized in Table 1. The median age of the cohort was 46 years, with a female predominance (63.4%). The majority of patients (80.2%) had a good performance status (ECOG 0–1). The classical variant was the dominant subtype (91.6%). The frequency of *Fms-like tyrosine kinase 3-internal tandem duplication* mutation (*FLT3-ITD*) and *FLT3-tyrosine kinase domain (TKD)* mutations was 15.3% and 5.3%, respectively. Risk stratification analysis indicated that 22.9% of the cohort was classified as high-risk according to the Sanz score. When the Österroos model was applied, 37.4% were categorized as high-risk and 10.7% as ultra-high-risk.

### 3.2. Distinct Clinicobiological Profiles of Early Mortality

Early mortality occurred in 33 patients (25.2%) within the first 30 days of induction. An analysis of the temporal distribution of mortality distinguished two specific subgroups: 8 patients (6.1%) experienced VED (<7 days), while the remaining 25 deaths (19.1%) occurred during the subsequent early period (7–30 days). The primary causes of VED were intracranial hemorrhage (n = 4), DIC-related multi-organ failure (n = 3) and sepsis (n = 1). During the subsequent early period, mortalities were attributed to sepsis (n = 10), DIC-related multi-organ failure (n = 5), fatal bleeding (n = 4), differentiation syndrome (n = 3), and thrombosis (n = 3). In contrast, late mortalities were exclusively driven by primary disease complications secondary to clinical relapse.

A detailed comparative analysis of clinical characteristics across these groups is presented in Table 2. Regarding demographic factors, patients experiencing mortality between days 7 and 30 were significantly older (median 59 years) compared to both the VED group (38.5 years) and survivors (41.5 years) (*p* < 0.001). Despite this age disparity, both early mortality cohorts (VED and 7–30 days) shared a phenotype of aggressive disease burden and poor functional status compared to long-term survivors. Patients in both mortality groups exhibited significantly poorer performance status (ECOG ≥ 2) and markedly elevated median levels of leukocytes, LDH and serum creatinine (*p* < 0.05). Similarly, the prevalence of *FLT3* mutations was significantly higher in both early death groups compared to survivors (*p* = 0.003). Compared to survivors, established risk stratification models consistently assigned a significantly higher proportion of patients in the early death groups to their respective highest-risk tiers (specifically, the high-risk category for the Sanz model and the high- and ultra-high-risk categories for the Österroos and Cai models). Regarding treatment-related complications, although the incidence of DS was numerically higher in the VED (25%) and 7–30-day mortality groups (16%) compared to survivors (10.2%), this difference did not reach statistical significance (*p* = 0.248).

**Figure 2:**
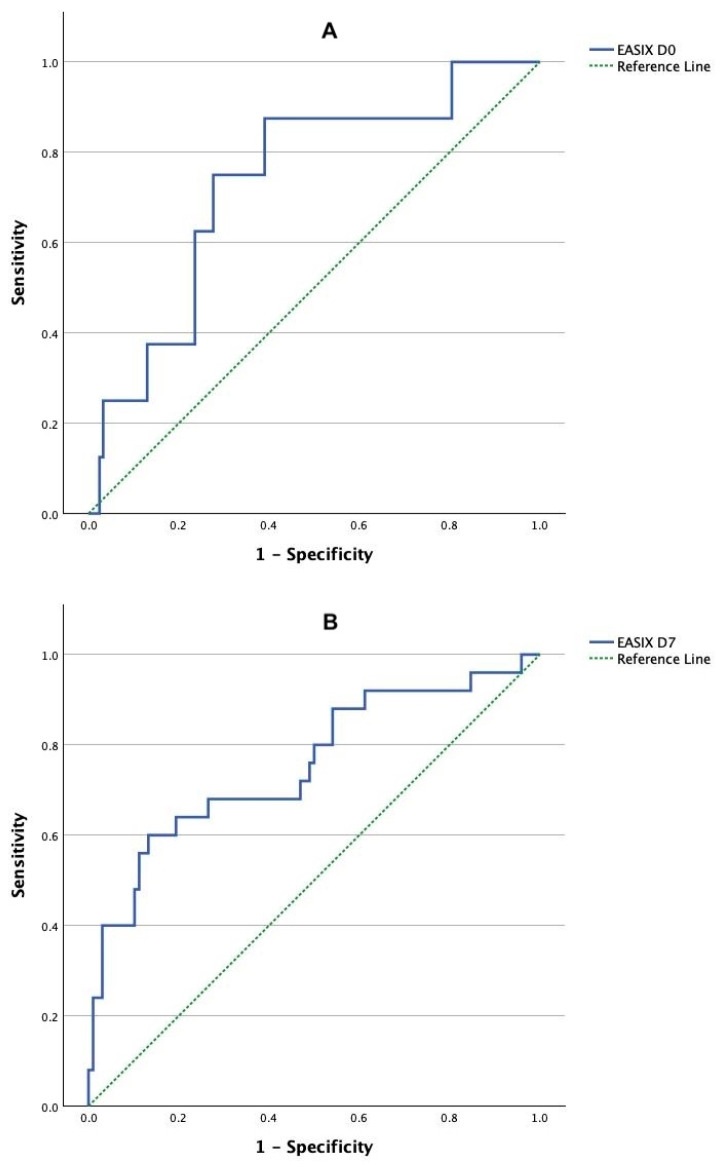
ROC curve analysis of baseline (**A**) and day 7 (**B**) EASIX scores for predicting early death.

A distinct temporal pattern was observed in endothelial stress markers. Baseline EASIX (D0) scores were significantly elevated in the VED group compared to the other cohorts (*p* = 0.015), with no significant difference observed between the 7–30-day mortality group and survivors at admission. Reflecting this, as illustrated in Figure 2, ROC analysis identified an EASIX D0 cut-off of 3.58 (AUC: 0.734; sensitivity: 87.5%; specificity: 61.0%; *p* = 0.007) as a specific predictor for fatal events occurring in the very early period (<7 days). In contrast, dynamic endothelial assessment was specifically analyzed for its ability to predict mortality occurring between days 7 and 30. In this landmark window, EASIX D7 and ΔEASIX scores significantly discriminated the mortality group from survivors (*p* < 0.001 and *p* = 0.020, respectively). Consequently, as demonstrated in Figure 2, an EASIX D7 cut-off of 2.46 (AUC: 0.754; sensitivity: 80.0%; specificity: 50.0%; *p* < 0.001) was identified as a significant predictor.

**Figure 3:**
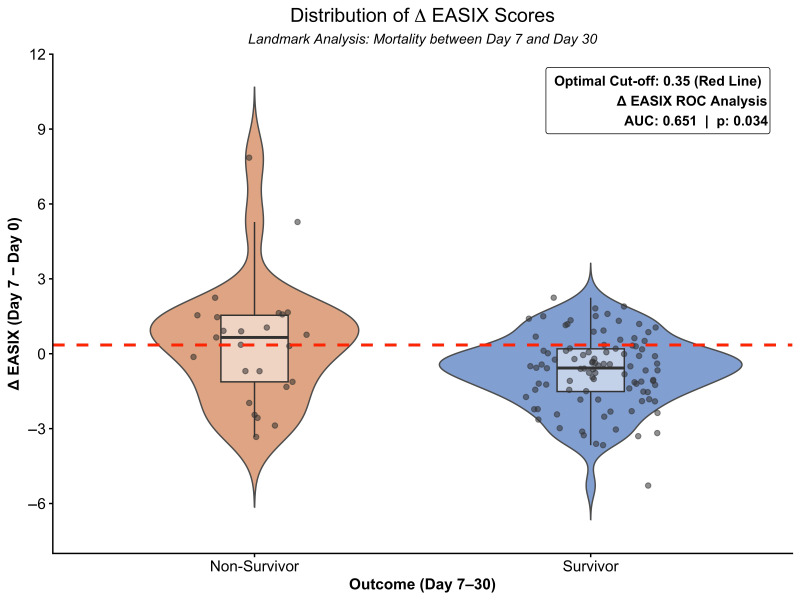
Distribution of dynamic EASIX changes (ΔEASIX) stratified by survival status in landmark period (day 7–30). Boxplots indicate medians and interquartile ranges (IQRs). Median ΔEASIX was significantly higher in non-survivors (0.65, IQR: −1.12 to 1.54) compared to survivors (−0.57, IQR: −1.52 to 0.20) (*p* = 0.020). Inset shows ROC analysis for optimal cut-off.

Furthermore, a worsening endothelial status—defined as an increase in the EASIX score (ΔEASIX)—was strongly predictive of mortality in the subsequent early period (7–30 days). As shown in Figure 3, non-survivors exhibited progressive deterioration, whereas survivors typically displayed a stable or improving course. Statistically, the optimal cut-off for this increase was determined as >0.35 (AUC: 0.651; sensitivity: 56.0%; specificity: 79.6%; *p* = 0.034); patients exceeding this threshold were significantly more likely to experience early mortality, while those maintaining values below this limit were predominantly survivors.

### 3.3. Dynamic Risk Stratification and Predictors of Early Mortality

**Figure 4:**
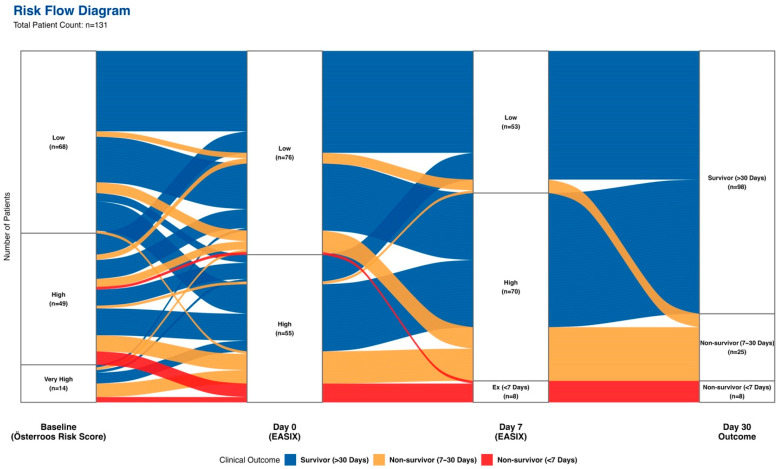
Sankey diagram of patient flow: from baseline risk and dynamic EASIX evolution to early mortality outcomes.

The prognostic impact of dynamic risk changes is visualized in a multi-stage Sankey diagram, as shown in Figure 4. Initial assessment revealed a discordance between static risk scores and biological endothelial stress. Among patients stratified as low-risk by the Österroos model, 22% already had high baseline EASIX scores (EASIX D0 > 3.58), whereas 36.5% of the high/ultra-high-risk strata had low initial EASIX scores. Crucially, the baseline Österroos model successfully identified the patients at the highest immediate risk: all eight VEDs occurred exclusively in the high- or ultra-high-risk groups. Longitudinal tracking exposed critical risk shifts that determined patient fate. While 48.5% (33/68) of the Österroos low-risk cohort deteriorated to a high EASIX status by day 7, notable plasticity was observed in the Österroos high-risk group. Specifically, 34.7% (17/49) of these patients successfully downstaged to a low-EASIX status. This biological recovery, termed the “Rescue Effect,” was associated with a marked reduction in mortality from 56.8% to 27.8%. Consequently, the integration of day 7 dynamic status provided a statistically significant improvement in risk reclassification for non-survivors (Net Reclassification Improvement, NRI+ = 0.500; *p* = 0.015).

As detailed in Table 3, multivariate logistic regression analysis was performed on the landmark cohort to identify independent predictors of early mortality. In this model, worsening endothelial function (ΔEASIX > 0.35) emerged as the strongest independent predictor, increasing the risk by 12.4-fold (OR: 12.41; 95% CI: 1.96–78.46; *p* = 0.007). The baseline Österroos risk score also retained independent significance (OR: 8.58; *p* = 0.002), confirming the complementary value of static and dynamic assessments.

### 3.4. Long-Term Survival Outcomes

**Figure 5:**
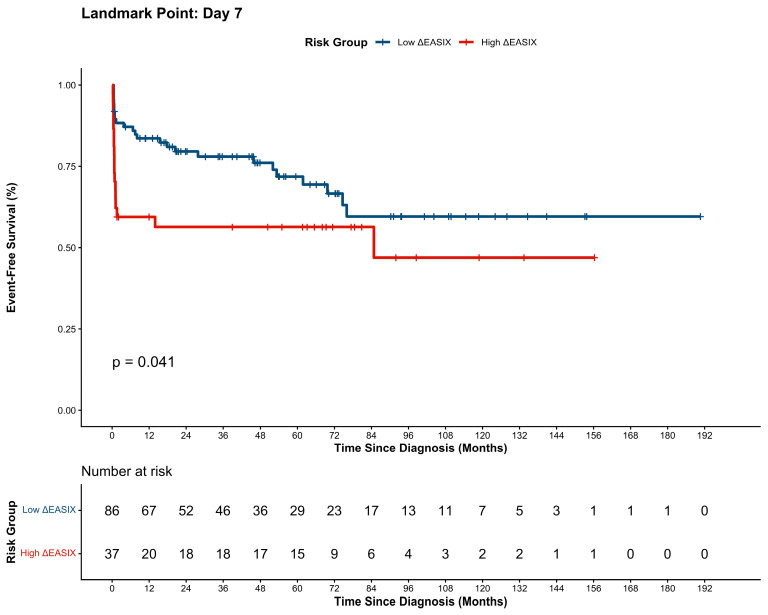
Landmark Kaplan–Meier analysis of EFS stratified by ΔEASIX status.

**Figure 6:**
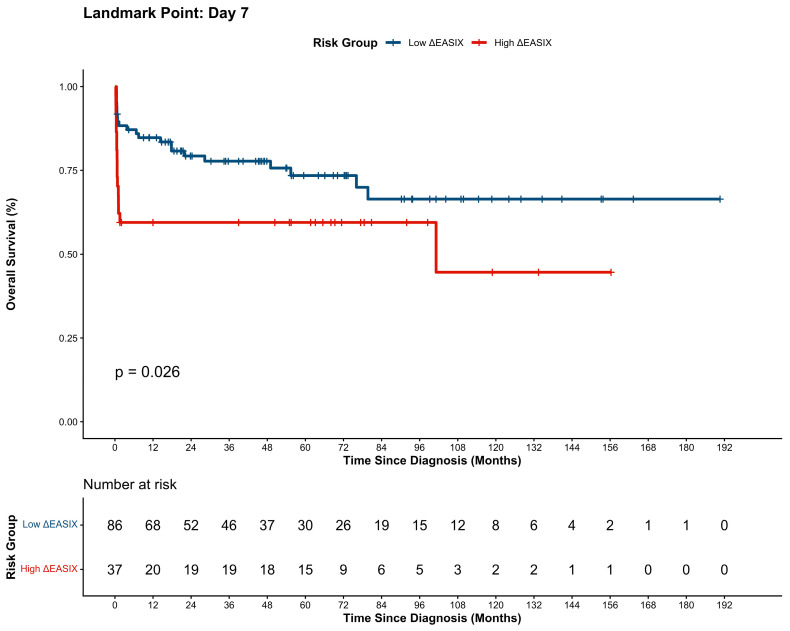
Landmark Kaplan–Meier analysis of OS stratified by ΔEASIX status.

The long-term prognostic impact of dynamic endothelial assessment was evaluated using a landmark analysis to mitigate immortal time bias. Kaplan–Meier survival curves, stratified by the kinetic trajectory of ∆EASIX (cut-off: >0.35), are illustrated in Figure 5 and Figure 6. Independent prognostic factors for these outcomes were identified via multivariate Cox proportional hazards regression models, as detailed in Table 4 and Table 5. With a median follow-up of 35.7 months, landmark analysis confirmed that a worsening endothelial status (high ∆EASIX) was associated with significantly inferior long-term outcomes. The low-∆EASIX cohort maintained robust EFS (1-year: 83.6%; 3-year: 77.8%), whereas the high-∆EASIX group exhibited markedly lower rates (1-year: 59.5%; 3-year: 56.3%; *p* = 0.041). A parallel disparity was evident in OS, where the low-∆EASIX group achieved superior 1- and 3-year rates (84.8% and 78.0%) compared to the high-∆EASIX cohort (59.5% and 59.5%; *p* = 0.026). Furthermore, consistent with these dynamic kinetic findings, elevated static day 7 EASIX scores were also predictive of inferior long-term survival, as detailed in Supplementary Figures S1 and S2.

In the multivariate analysis, a worsening endothelial status (ΔEASIX > 0.35) was an independent predictor of both poor EFS (HR: 5.70; 95% CI: 1.72–18.82; *p* = 0.004) and poor OS (HR: 3.69; 95% CI: 1.19–11.40; *p* = 0.023). Similarly, the baseline Österroos risk score (>low) maintained its independent significance for both EFS (HR: 8.58; *p* = 0.002) and OS (HR: 7.29; *p* = 0.032). Notably, consistent with the early mortality analyses, traditional static risk factors such as FLT3 mutation status and Sanz score lost their independent prognostic value when adjusted for dynamic endothelial markers in the long-term models.

## 4. Discussion

Current risk stratification models for early death and relapse in APL rely exclusively on static baseline parameters [6,14,29], failing to capture the intense biological volatility of the induction phase. Given the rapid and profound physiological shifts characteristic of this distinct AML subtype, such static assessments inevitably lead to significant risk misclassification. Consequently, despite remarkable therapeutic advances, early death remains a persistent clinical challenge [34], necessitating markers that reflect real-time physiological evolution. To the best of our knowledge, this is the first study to evaluate the dynamic evolution of EASIX score during induction therapy and its prognostic impact in patients with APL. Our findings demonstrate that APL is not a static disease; rather, it follows a rapidly shifting clinicobiological trajectory determined by the host’s endothelial response to the leukemic insult and induction therapy. We identified that while baseline scores are valuable for predicting immediate fatalities (<7 days), the “kinetic trajectory” of endothelial stress is the decisive factor for subsequent outcomes. Specifically, a worsening endothelial status—defined as an increase in ΔEASIX (>0.35)—emerged as a robust, independent predictor across the entire prognostic spectrum, significantly correlating with early mortality (between days 7 and 30), inferior EFS and poor OS. Conversely, we described a “Rescue Effect,” in which high-risk patients who achieved endothelial stabilization by day 7 demonstrated significantly improved survival, highlighting the critical importance of dynamic risk reclassification.

Despite the curative potential of current regimens, ED persists as the primary barrier to survival. While clinical trials report ED rates as low as 3–6%, real-world registries consistently document significantly higher rates ranging from 17% to 29% [34,35]. Our cohort confirmed this real-world disparity with an ED rate of 25.2% and VED rate of 6.1%. Beyond the relapse-oriented Sanz score, contemporary risk models developed by Österroos and Cai have specifically targeted early mortality by incorporating key variables such as age, leukocytosis, thrombocytopenia, and elevated LDH [6,14]. Regarding immediate mortality, Infante et al. reported a VED rate of 4.8% and identified serum creatinine as a significant independent predictor of death within the first 7 days [10]. Additionally, other independent studies have consistently highlighted poor ECOG performance status as a critical determinant of early death [9]. Crucially, in the modern era where late relapses are infrequent, long-term survival is mathematically determined by the ability to survive the induction phase [1,36]. Consequently, clinical markers that successfully predict early mortality—such as poor ECOG status and Österroos score—also emerged as significant predictors for EFS and OS in our analysis. Serving inherently as a composite marker of vascular stress, EASIX demonstrates a robust capacity to predict both early fatal events and long-term survival. In this context, the discordance between Österroos and baseline EASIX scores in our Sankey diagram further highlights their distinct pathophysiological targets. While Österroos reflects leukemic burden and demographic risk, EASIX specifically quantifies host endothelial vulnerability. Thus, a patient may be high-risk according to Österroos (e.g., hyperleukocytosis) but have a low EASIX if systemic endothelial dysfunction has not ensued or vice versa. Clinically, these scores are highly complementary, a relationship robustly corroborated by our multivariate models, where both independently retained prognostic significance. Integrating dynamic EASIX allows clinicians to identify hidden high-risk patients experiencing profound endothelial dysfunction despite a seemingly favorable traditional profile.

APL is defined by a “double-hit” mechanism involving concomitant primary hyperfibrinolysis and DIC, standing in sharp contrast to sepsis-associated coagulopathy, which is typically characterized by fibrinolytic shutdown [37,38]. This coagulopathy is driven not only by the overexpression of Annexin A2 and tissue-type plasminogen activator (t-PA), which generate excessive plasmin, but also by the release of tissue factor (TF) and cancer procoagulant (CP) from leukemic promyelocytes, leading to profound thrombin generation [38,39,40,41,42]. Furthermore, the release of inflammatory cytokines induces the expression of adhesion molecules on the vascular endothelium, promoting leukemic cell sequestration and microvascular injury [42,43]. Since this biological storm is fluid, static snapshots often fail to capture the escalating risk. Indeed, Infante et al. demonstrated that worsening ISTH DIC score over time was a stronger predictor of mortality than baseline values, validating the concept that the evolution of coagulopathy is the critical determinant of survival [11]. Importantly, while APL-associated early mortality is clinically heterogeneous in its direct causes ranging from catastrophic hemorrhage and severe differentiation syndrome to fulminant sepsis, the underlying pathophysiology consistently converges on profound systemic endothelial dysfunction and microvascular injury. EASIX has previously been validated as a predictor of endothelial-driven complications, including sinusoidal obstruction syndrome (VOD), graft-versus-host disease (GVHD), and chimeric antigen receptor T-cell (CAR-T) toxicity [20,32,33]. As a result, dynamic EASIX serves as a robust predictor of all-cause early mortality, capturing this shared terminal pathway of systemic vascular collapse rather than being limited exclusively to hemorrhagic events. Within this unique pathophysiological framework, the individual components of EASIX directly mirror the severity of APL-induced vascular collapse: elevated LDH reflects profound tissue ischemia and leukemic cell turnover; thrombocytopenia represents the exhaustive consumptive coagulopathy; and rising creatinine signifies acute microvascular hypoperfusion and capillary leak. In this manner, their combined kinetic trajectory precisely tracks the clinical deterioration leading to mortality.

Induction therapy further complicates this vascular landscape. While ATRA is essential for differentiation, it triggers the release of inflammatory cytokines and induces neutrophil extracellular traps (NETosis), which can aggravate endothelial injury and increase the risk of differentiation syndrome in the initial phase [42,44]. Conversely, recent evidence suggests that ATO may exert an endothelial-protective effect, downregulating adhesion molecules and reducing vascular permeability compared to chemotherapy-based regimens [45,46]. In our study, which used the AIDA protocol without upfront ATO, the dynamic elevation of EASIX likely captured the cumulative endothelial insult from leukemic hyperfibrinolysis, chemotherapy-induced toxicity and ATRA-mediated NETosis. This suggests that dynamic endothelial monitoring is particularly vital in settings where chemotherapy remains the backbone of induction. However, given that the ATRA plus ATO combination is now the standard of care for low-to-intermediate-risk APL, a critical question is whether the predictive value of ΔEASIX remains consistent in chemotherapy-free settings. While frontline ATO-based regimens have dramatically improved long-term survival and reduced relapse rates, they have not completely abrogated early mortality, which still occurs in approximately 7–10% of patients, even in recent clinical trials [35,36]. Because these early fatal events remain fundamentally driven by the inherent leukemic coagulopathy, severe differentiation syndrome and DIC-related multi-organ failure, we hypothesize that the dynamic prognostic utility of ΔEASIX will remain highly relevant in ATO-treated patients. Nevertheless, given the disparate endothelial effects of these agents, the optimal prognostic thresholds of the ΔEASIX score may differ from those established in chemotherapy-treated cohorts.

Our study has several limitations. First, the retrospective, two-center design and modest sample size precluded the evaluation of intermediate time points (e.g., days 3 and 14) for a more detailed kinetic assessment of the EASIX score. Second, while the day 7 landmark analysis was methodologically necessary to mitigate immortal time bias, it inherently excluded very early deaths, introducing an unavoidable selection bias. Third, since platelet count is a component of the EASIX formula, daily platelet transfusions can potentially affect the score. Although patients received standardized supportive care, our retrospective design prevented adjusting for exact transfusion volumes. Fourth, although the absolute number of early deaths is consistent with comparable real-world APL cohorts, this limited number of events naturally restricts the statistical power of our multivariate models. While stringent stepwise selection methods were utilized to minimize overfitting, the independent prognostic factors identified herein require prospective external validation. Finally, since our cohort was restricted to patients treated with the AIDA protocol, future studies are warranted to confirm the prognostic utility of dynamic EASIX monitoring in chemotherapy-free, ATO-based regimens.

## 5. Conclusions

In conclusion, our study is the first to demonstrate that dynamic endothelial stress evolution is a critical prognostic factor in APL. We identified that worsening endothelial status (ΔEASIX > 0.35) is a robust, independent predictor of early mortality and significantly stratifies patients for long-term survival. By capturing the physiological volatility of induction that static biomarkers miss, dynamic EASIX monitoring offers a practical tool for real-time risk refinement. Clinically, a high ΔEASIX trajectory at day 7 serves as a “red flag” requiring immediate preemptive action. For these high-risk patients, clinicians should prioritize stricter transfusion thresholds to counteract profound coagulopathy, maintain rigorous surveillance for severe infections like sepsis and optimize ongoing prophylactic measures for differentiation syndrome. Conversely, patients with stable endothelial function by day 7 may safely proceed with standard care. Furthermore, given the impact of ATRA-induced NETosis on endothelial damage, this high-risk cohort is an ideal target population for evaluating emerging therapies aimed at preventing NETosis. Ultimately, further large-scale, prospective, multicenter studies are required to validate these findings and establish the clinical utility of this dynamic model across different therapeutic protocols.

## Figures and Tables

**Table 1:** Baseline clinical and demographic characteristics (n = 131).

Parameters	n (%)
**Age, years**	
Median (Range)	46 (18–82)
<50 years	78 (59.5)
≥70 years, (%)	8 (6.1)
**Female Sex**	83 (63.4)
**Comorbidities**	
Diabetes Mellitus	19 (14.5)
Hypertension	25 (19.1)
Coronary Artery Disease	3 (2.3)
Chronic Obstructive Pulmonary Disease	2 (1.5)
**ECOG Performance Status**	
0–1	105 (80.2)
≥2	26 (19.8)
**Pathological Subtype**	
Classical	120 (91.6)
Hypogranular variant	11 (8.4)
***PML::RARA*** **Transcript**	
BCR1	111 (84.7)
BCR2	4 (3.1)
BCR3	16 (12.2)
***FLT3*** **Mutation Status**	
ITD	20 (15.3)
TKD	7 (5.3)
**ISTH Risk Score**	
<5	16 (12.2)
≥5	115 (87.8)
**Sanz Risk Score**	
Low risk	60 (45.8)
Intermediate risk	41 (31.3)
High risk	30 (22.9)
**Österroos Risk Score**	
Low risk	68 (51.9)
High risk	49 (37.4)
Ultra-high risk	14 (10.7)
**Cai Risk Score**	
Low risk	49 (37.4)
Intermediate risk	41 (31.3)
High risk	32 (24.4)
Ultra-high risk	9 (6.9)
**Differentiation Syndrome**	16 (12.2)
**Early Death**	33 (25.2)
Very early death (<7 days)	8 (6.1)
Subsequent early death (7–30 days)	25 (19.1)
**Disease Relapse**	13 (9.9)
**Total Number of Deaths**	46 (35.1)

ECOG: Eastern Cooperative Oncology Group; *PML::RARA*: Promyelocytic leukemia–Retinoic acid receptor alpha fusion gene; BCRs: Breakpoint cluster regions; *FLT3*: Fms-like tyrosine kinase 3; ITD: Internal tandem duplication mutation; TKD: Tyrosine kinase domain mutation; ISTH: International Society of Thrombosis and Haemostasis.

**Table 2:** Comparison of clinical characteristics and risk scores according to early mortality status.

Parameters	Very Early Death (<7 Days) n:8 (%)	Early Death (7–30 Days) n:25 (%)	30-Day Survivors n:98 (%)	*p *-Value
**Age, years (median)**	38.5 (20–75)	59 (28–82)	41.5 (18–74)	**<0.001 ^k^**
**Female Sex**	6 (75)	15 (60)	62 (63.3)	0.806 ^χ2^
**ECOG PS**				
0–1	2 (25)	10 (40)	93 (94.8)	**<0.001** ^χ2^
≥2	6 (75)	15 (60)	5 (5.2)	
**Pathological Subtype**				
Classical	7 (87.5)	24 (96)	89 (90.8)	0.508 ^χ2^
Hypogranular variant	1 (12.5)	1 (4)	9 (9.2)	
***PML::RARA*** **Transcript**				
BCR1	7 (87.5)	24 (96)	80 (81.6)	0.528 ^χ2^
BCR2	-	-	4 (4.1)	
BCR3	1 (12.5)	1 (4)	14 (14.3)	
***FLT3*** **Mutation Status**	4 (50)	9 (36)	14 (14.3)	**0.003** ^χ2^
**Total Leukocytes, 10^9^/L (median)**	85.6 (12.6–132)	5.4 (0.49–161.6)	1.8 (0.13–131.3)	**<0.001 ^k^**
**Hemoglobin, g/dL (mean ± SD)**	8.48 ± 1.09	9.34 ± 1.86	9.64 ± 2.2	0.298 ^a^
**Platelets, 10^9^/L (median)**	46.8 (13–90)	30.9 (4.9–125)	28 (2.6–173)	0.144 ^k^
**Serum LDH, IU/L (median)**	898 (481–1318)	522 (168–3245)	289 (107–1386)	**<0.001 ^k^**
**Serum Creatinine, mg/dL**	0.92 (0.7–1.6)	0.96 (0.6–1.4)	0.74 (0.4–1.2)	**<0.001 ^k^**
**CRP, mg/L (median)**	18.3 (7.1–177)	22.6 (0.9–272)	5.5 (0.1–302)	**0.012 ^k^**
**ISTH Risk Score**				
<5	1 (12.5)	3 (12)	12 (12.2)	>0.999 ^χ2^
≥5	7 (87.5)	22 (88)	86 (87.8)	
**Sanz Risk Score**				
Low risk	-	8 (32)	52 (53.1)	**<0.001 ^χ2^**
Intermediate risk	2 (25)	7 (28)	32 (32.7)	
High risk	6 (75)	10 (40)	14 (14.2)	
**Österroos Risk Score**				
Low risk	-	7 (28)	61 (62.2)	**<0.001 ^χ2^**
High risk	6 (75)	12 (48)	31 (31.6)	
Ultra-high risk	2 (25)	6 (24)	6 (6.2)	
**Cai Risk Score**				
Low risk	-	3 (12)	46 (46.9)	
Intermediate risk	1 (12.5)	9 (36)	31 (31.6)	**<0.001 ^χ2^**
High risk	5 (62.5)	7 (28)	20 (20.4)	
Ultra-high risk	2 (25)	6 (24)	1 (1.1)	
**EASIX D0 (median)**	4.23 (1.91–4.55)	3.65 (0.38–8.21)	3.13 (0.94–7.02)	**0.015 ^k^**
**EASIX D7 (median)**	-	3.88 (0.21–10.55)	2.47 (0.05–6.8)	**<0.001 ^m^**
**∆EASIX**	-	0.65 (−3.33–7.85)	−0.57 (−5.28–2.24)	**0.020 ^m^**
**Differentiation Syndrome**	2 (25)	4 (16)	10 (10.2)	0.248 ^χ2^

ECOG PS: Eastern Cooperative Oncology Group Performance status; *PML::RARA*: Promyelocytic leukemia–Retinoic acid receptor alpha fusion gene; BCRs: Breakpoint cluster regions; *FLT3*: Fms-like tyrosine kinase 3; LDH: Lactate dehydrogenase; CRP: C-reactive protein; ISTH: International Society of Thrombosis and Haemostasis; EASIX D0: Baseline endothelial activation and stress index at admission; EASIX D7: Endothelial activation and stress index on day 7; ∆EASIX: Difference between the day 7 and admission EASIX scores. ^k^: Kruskal–Wallis test; ^χ2^: Chi-squared test; ^a^: One-way ANOVA test; ^m^: Mann–Whitney U test. Bold values: Statistically significant.

**Table 3:** Predictors of early mortality between day 7 and day 30: landmark logistic regression analysis.

Factor ^a^	Univariate Analysis				Multivariate Analysis			
	OR	95% CI		*p*-Value	OR	95% CI		*p*-Value
		Lower	Upper			Lower	Upper	
Sex (Female)	0.871	0.354	2.141	0.763				
ECOG PS (≥2)	5.095	1.668	15.564	**0.004**				
Pathological Subtype (Variant)	2.427	0.293	20.110	0.411				
*PML::RARA* Transcript (BCR3)	0.250	0.031	1.999	0.191				
*FLT3* Mutation	8.143	1.923	34.478	**0.004**				
Comorbidities	2.604	0.954	7.108	0.062				
CRP (mg/L)	1.005	0.998	1.013	0.173				
ISTH Risk Score	1.220	0.842	1.768	0.293				
Sanz Risk Score (>Low)	2.135	1.213	3.760	**0.009**				
Differentiation Syndrome	1.676	0.479	5.871	0.419				
Österroos Risk Score (>Low)	3.026	1.563	5.857	**0.001**	8.586	2.255	32.694	**0.002**
Cai Risk Score (>Intermediate)	2.928	1.711	5.009	**<0.001**				
EASIX D0 (High)	1.950	0.803	4.733	0.140				
EASIX D7 (High)	3.840	1.334	11.050	**0.013**				
∆EASIX (High)	5.182	2.044	13.137	**<0.001**	12.409	1.962	78.462	**0.007**

OR: Odds ratio; CI: Confidence interval; ECOG PS: Eastern Cooperative Oncology Group Performance status; *PML::RARA*: Promyelocytic leukemia–Retinoic acid receptor alpha fusion gene; BCRs: Breakpoint cluster regions; *FLT3*: Fms-like tyrosine kinase 3; CRP: C-reactive protein; ISTH: International Society of Thrombosis and Haemostasis; EASIX D0: Baseline endothelial activation and stress index at admission; EASIX D7: Endothelial activation and stress index on day 7; ∆EASIX: Difference between the day 7 and admission EASIX scores. ^a^: The analysis was performed on the landmark cohort (n = 123), excluding patients with very early death (<7 days). Bold values: Statistically significant.

**Table 4:** Univariate and multivariate landmark Cox regression analyses for EFS.

Factor ^a^	Univariate Analysis				Multivariate Analysis			
	HR	95% CI		*p*-Value	HR	95% CI		*p*-Value
		Lower	Upper			Lower	Upper	
Sex (Female)	0.784	0.425	1.444	0.435				
ECOG PS (≥2)	4.165	2.171	7.992	**<0.001**	3.094	1.158	8.264	**0.024**
Pathological Subtype (Variant)	1.026	0.365	2.882	0.961				
*PML::RARA* Transcript (BCR3)	0.527	0.163	1.708	0.286				
*FLT3* Mutation	3.840	1.561	9.448	**0.003**				
Comorbidities	1.687	0.828	3.440	0.150				
CRP (mg/L)	1.002	0.997	1.007	0.462				
ISTH Risk Score	1.063	0.834	1.353	0.623				
Sanz Risk Score (>Low)	1.603	1.090	2.357	**0.016**				
Differentiation Syndrome	1.870	0.827	4.229	0.133				
Österroos Risk Score (>Low)	2.345	1.530	3.592	**<0.001**	8.584	2.268	32.487	**0.002**
Cai Risk Score (>Intermediate)	2.150	1.531	3.018	**<0.001**				
EASIX D0 (High)	1.724	0.938	3.169	0.080				
EASIX D7 (High)	2.319	1.185	4.535	**0.014**				
∆EASIX (High)	1.880	1.013	3.488	**0.045**	5.703	1.727	18.824	**0.004**

HR: Hazard ratio; CI: Confidence interval; ECOG PS: Eastern Cooperative Oncology Group Performance status; *PML::RARA*: Promyelocytic leukemia–Retinoic acid receptor alpha fusion gene; BCRs: Breakpoint cluster regions; *FLT3*: Fms-like tyrosine kinase 3; CRP: C-reactive protein; ISTH: International Society of Thrombosis and Haemostasis; EASIX D0: Baseline endothelial activation and stress index at admission; EASIX D7: Endothelial activation and stress index on day 7; ∆EASIX: Difference between the day 7 and admission EASIX scores. ^a^: The analysis was performed on the landmark cohort (n = 123), excluding patients with very early death (<7 days), Bold values: Statistically significant.

**Table 5:** Univariate and multivariate landmark Cox regression analysis for OS.

Factor ^a^	Univariate Analysis				Multivariate Analysis			
	HR	95% CI		*p*-Value	HR	95% CI		*p*-Value
		Lower	Upper			Lower	Upper	
Sex (Female)	0.824	0.433	1.571	0.557				
ECOG PS (≥2)	4.393	2.275	8.484	**<0.001**	2.888	1.086	7.679	**0.034**
Pathological Subtype (Variant)	1.279	0.391	4.179	0.684				
*PML::RARA* Transcript (BCR3)	0.579	0.178	1.884	0.364				
*FLT3* Mutation	5.092	1.899	13.653	**0.001**				
Comorbidities	1.664	0.785	3.525	0.184				
CRP (mg/L)	1.002	0.997	1.008	0.395				
ISTH Risk Score	1.062	0.824	1.370	0.641				
Sanz Risk Score (>Low)	1.762	1.180	2.631	**0.006**				
Differentiation Syndrome	2.050	0.899	4.675	0.088				
Österroos Risk Score (>Low)	2.503	1.611	3.889	**<0.001**	7.296	1.181	45.067	**0.032**
Cai Risk Score (>Intermediate)	2.409	1.684	3.445	**<0.001**				
EASIX D0 (High)	1.659	0.875	3.143	0.121				
EASIX D7 (High)	2.231	1.106	4.504	**0.025**				
∆EASIX (High)	2.042	1.071	3.894	**0.030**	3.697	1.199	11.402	**0.023**

HR: Hazard ratio; CI: Confidence interval; ECOG PS: Eastern Cooperative Oncology Group Performance status; *PML::RARA*: Promyelocytic leukemia-Retinoic acid receptor alpha fusion gene; BCRs: Breakpoint cluster regions; *FLT3*: Fms-like tyrosine kinase 3; CRP: C-reactive protein; ISTH: International Society of Thrombosis and Haemostasis; EASIX D0: Baseline endothelial activation and stress index at admission; EASIX D7: Endothelial activation and stress index on day 7; ∆EASIX: Difference between the day 7 and admission EASIX scores. ^a^: The analysis was performed on the landmark cohort (n = 123), excluding patients with very early death (<7 days). Bold values: Statistically significant.

## Data Availability

The raw data supporting the conclusions of this article will be made available by the authors on request.

## Supplementary Materials

The following supporting information can be down-loaded at https://www.mdpi.com/article/10.3390/cancers18050843/s1, Figure S1: Landmark Kaplan-Meier analysis of EFS stratified by day 7 EASIX status; Figure S2: Landmark Kaplan-Meier analysis of OS stratified by day 7 EASIX status.
