# Supplementary material for: The Dynamic Endothelial Activation and Stress Index (EASIX) as a Predictor of Early Death and Long-Term Survival in Acute Promyelocytic Leukemia (APL): A Multicenter Study
Source: Cancers (Basel). 2026 Mar 5;18(5):843. doi: 10.3390/cancers18050843 (PMC12984233; doi:10.3390/cancers18050843)
Supplement: Supplementary file 1 [file cancers-18-00843-s001.zip › cancers-4153901-supplementary.pdf]

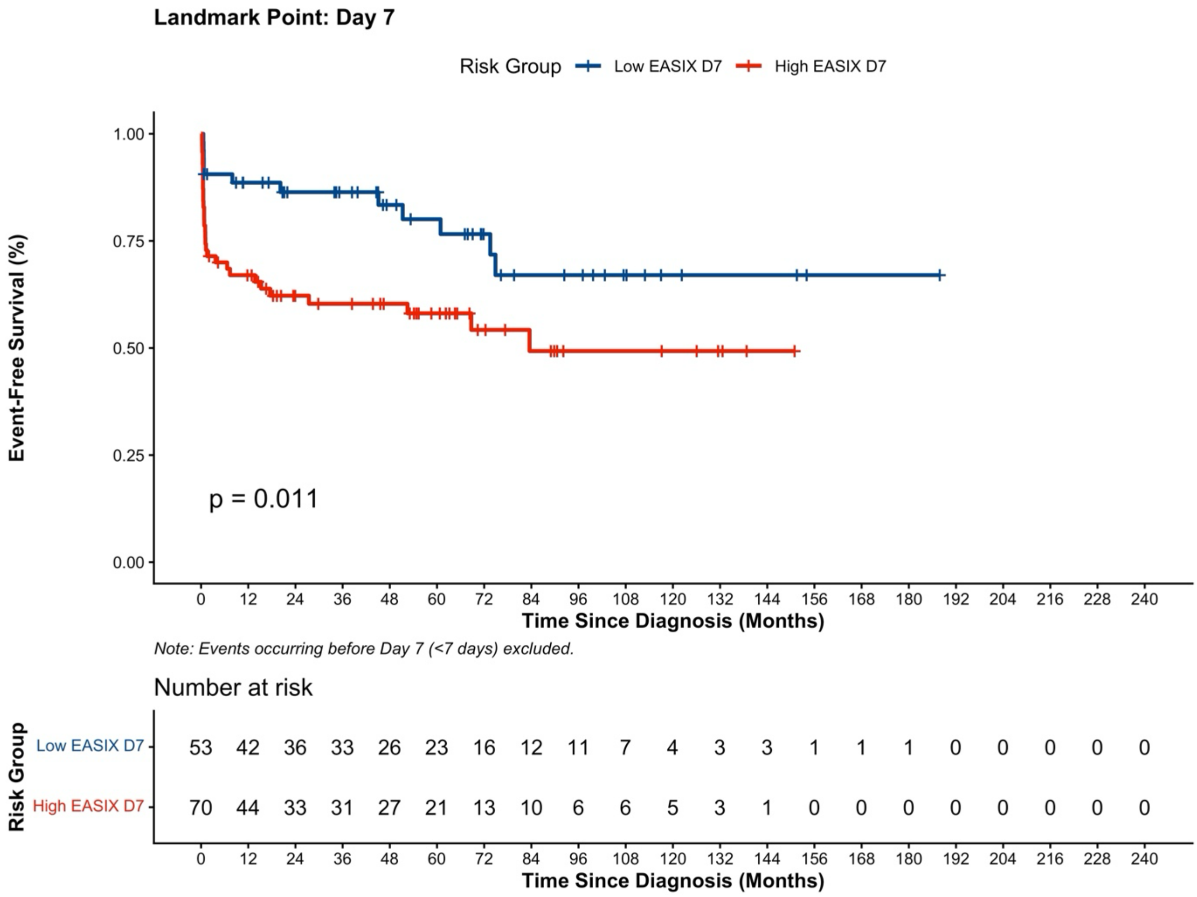

Supplementary Figure S1. Landmark Kaplan-Meier analysis of EFS stratified by day 7 EASIX status.

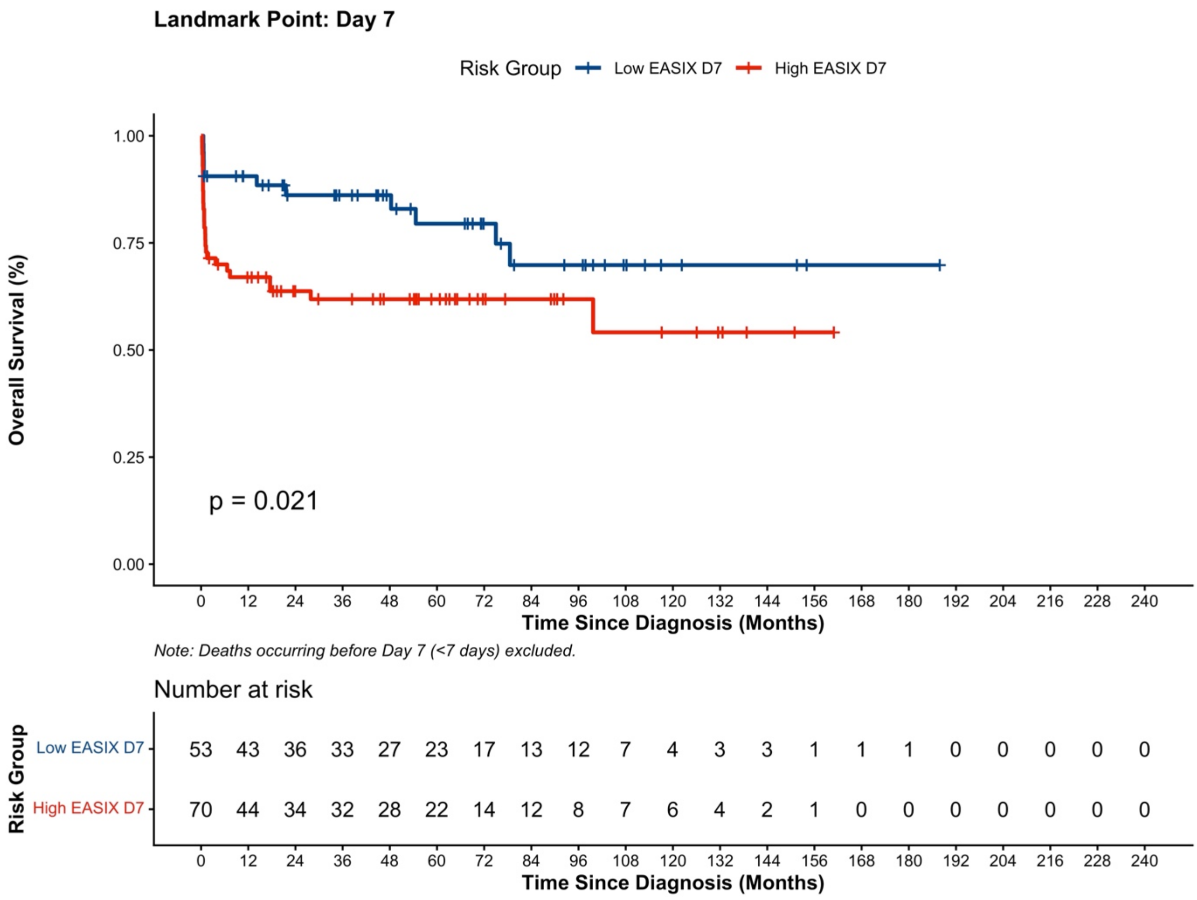

Supplementary Figure S2. Landmark Kaplan-Meier analysis of OS stratified by day 7 EASIX status.
